# Functionally Relevant Domains of the Prion Protein Identified *In Vivo*

**DOI:** 10.1371/journal.pone.0006707

**Published:** 2009-09-07

**Authors:** Frank Baumann, Jens Pahnke, Ivan Radovanovic, Thomas Rülicke, Juliane Bremer, Markus Tolnay, Adriano Aguzzi

**Affiliations:** 1 Institute of Neuropathology, University Hospital of Zurich, Zurich, Switzerland; 2 Department for Cellular Neurology, Hertie Institute of Clinical Brain Research, Tübingen, Germany; 3 Institute of Laboratory Animal Science and Biomodels Austria, University of Veterinary Medicine Vienna, Vienna, Austria; Mental Health Research Institute of Victoria, Australia

## Abstract

The prion consists essentially of PrP^Sc^, a misfolded and aggregated conformer of the cellular protein PrP^C^. Whereas PrP^C^ deficient mice are clinically healthy, expression of PrP^C^ variants lacking its central domain (PrP_ΔCD_), or of the PrP-related protein Dpl, induces lethal neurodegenerative syndromes which are repressed by full-length PrP. Here we tested the structural basis of these syndromes by grafting the amino terminus of PrP^C^ (residues 1–134), or its central domain (residues 90–134), onto Dpl. Further, we constructed a soluble variant of the neurotoxic PrP_ΔCD_ mutant that lacks its glycosyl phosphatidyl inositol (GPI) membrane anchor. Each of these modifications abrogated the pathogenicity of Dpl and PrP_ΔCD_ in transgenic mice. The PrP-Dpl chimeric molecules, but not anchorless PrP_ΔCD_, ameliorated the disease of mice expressing truncated PrP variants. We conclude that the amino proximal domain of PrP exerts a neurotrophic effect even when grafted onto a distantly related protein, and that GPI-linked membrane anchoring is necessary for both beneficial and deleterious effects of PrP and its variants.

## Introduction

PrP^Sc^ is the main constituent of prions [1], the infectious agents causing transmissible spongiform encephalopathies (TSE). PrP^Sc^ is an aggregated and misfolded isoform of the cellular prion protein PrP^C^ [2] which is expressed in a broad range of tissues of most vertebrates [3]. Nascent PrP^C^ is exported to the lumen of the endoplasmic reticulum, deprived of its amino terminal signal sequence, glycosylated at two asparagine residues, and endowed with a GPI moiety which anchors it to the outer cell surface. Ablation of the *Prnp* gene, which encodes PrP^C^, abrogates prion replication [4] and toxicity [5]. *Prnp* ^o/o^ mice enjoy a normal life expectancy [6], but suffer from subtle neurological phenotypes [7] whose molecular basis has remained elusive [8].

**Figure 1:**
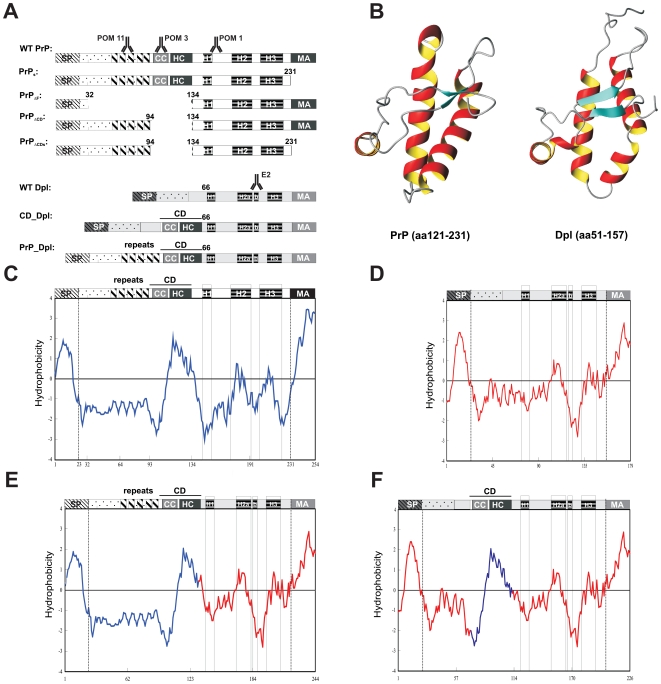
PrP and Dpl genes, chimeric constructs, and transgenic mice. (A) Schematic drawing of the deletion mutants utilized for generation of transgenic mice, and comparison to full-length wild-type PrP^C^ and Dpl. (B) Comparison of the structures of the globular carboxy terminal domains of murine PrP (left) and Dpl (right) (C–F) Hydrophobicity plots of wild-type murine PrP (C); wild-type murine Dpl (D); PrP_Dpl (E) and CD_Dpl (F). PrP and Dpl sequences are drawn in blue and red, respectively. SP: secretory signal peptide, cleaved after sorting of the precursor to endoplasmic reticulum. repeats: five repeats of eight amino acids. CC: charge cluster. HC: hydrophobic core. CD: central domain. H1–3: α-helix 1, 2, 3 of the globular carboxy proximal domain. MA: membrane anchor of precursor protein, replaced during maturation with glycosyl phosphatidyl inositol anchor. The symbol ▒ indicates the epitopes recognized by the monoclonal mouse antibodies POM1, POM3, POM11, and E2.

Transgenic expression of amino proximally truncated PrP^C^ mutants (PrP_ΔCD_, PrP_ΔE_ and PrP_ΔF_, henceforth collectively termed ΔPrP) causes early-onset ataxia and white-matter degeneration (Fig. 1A). Toxicity appears to correlate with partial or complete deletions of the conserved PrP central domain (CD, residues 94–134) [9], [10], [11] which bridges the flexible amino proximal tail and the globular carboxy proximal domain [12].

Another neurotoxic phenotype was detected in compound-heterozygous *Prnp* ^o/ZHII^ mice and in homozygous *Prnp* ^ZHII/ZHII^ mice [13] whose *Prnp* ^ZHII^ allele leads to ectopic expression of the PrP^C^-related protein Dpl [14], [15], [16], [17]. Neuronal expression of Dpl in Tg(Dpl) or Tg(N-Dpl) mice induces ataxia within 40–60 days [18], [19]. Despite 80% amino acid sequence dissimilarities [14], the overall 3D structure of Dpl is similar to that of PrP^C^ (Fig. 1B) and includes an unstructured amino proximal tail, a globular three-helix domain [20], and a GPI anchor. However, Dpl is physiologically not expressed in the adult nervous system [21] and, importantly, lacks any sequences comparable to the CD. Therefore, Dpl resembles the neurotoxic ΔPrP mutants. What is more, the toxicity of both Dpl and ΔPrP is counteracted by co-expression of full-length PrP^C^ [9], [10], [18], [22], [23], implying that it exploits common molecular pathways.

We reported previously that the removal of just the CD domain confers dramatic neurotoxicity to PrP. This suggests that the toxicity of Dpl may also result from the absence of a CD-like domain. Here, we tested this hypothesis by transgenic expression of two chimeric proteins, PrP_Dpl (residues 1–65 of Dpl replaced by residues 1–133 of PrP) and CD_Dpl (residues 90–133 of PrP inserted between residues 65 and 66 of Dpl). Transgenic mice expressing these proteins did not develop any clinical phenotypes. Additionally, coexpression of PrP_Dpl or of CD_Dpl ameliorated the clinical syndromes and prolonged the life expectancy of mice expressing neurotoxic ΔPrP mutants, in agreement with a previous report [24]. Since PrP is thought to be involved in signal transduction, we tested whether the toxicity of CD-deficient PrP mutants (PrP_ΔCD_) may require localization to membrane lipid rafts. Indeed, removal of the GPI addition signal from PrP_ΔCD_ prevents its neurotoxic effects.

## Results

### Transgenic mice expressing chimeric PrP-Dpl proteins and PrP_ΔCDs_

All chimeric mutants of Dpl and PrP described here are based on the ‘half-genomic’ pPrPHG backbone [25] whose expression pattern has been recently studied in detail [26]. This construct contains a redacted murine *Prnp* gene which lacks intron #2 and is flanked by 6 and 2.2 kb of 5′ and 3′ genomic regions, respectively. Neuronal expression of Dpl leads to ataxia, neuronal loss and demyelinating neuropathy [17], [18] [19] [[22]] while most of the toxicity of truncated PrP can be assigned to the lack of the central domain CD (residues 94–134) [10]. If the absence of a CD-like domain were responsible for its toxicity, addition of domains containing the CD region of PrP might detoxify Dpl.

We constructed CD_Dpl, a chimeric fusion protein consisting of codons 90–133 of mouse *Prnp* inserted between codons 65 and 66 of *Prnd* (Fig. 1A, F). This particular insertional position was chosen because hydrophobicity comparisons suggested that the resulting chimeric protein would resemble wild-type PrP (Fig. 1C–D). In a second construct termed PrP_Dpl, the amino terminus of PrP comprising codons 1–133 was fused to the carboxy terminus of Dpl comprising codons 66–179 (Fig. 1A; E). Pronuclear injection was performed into *Prnp* ^+/o^ zygotes resulting from a cross between *Prnp* ^o/o^ and wild-type (wt) C57BL/6N mice giving rise to transgenic founders on a *Prnp* ^+/o^ background (henceforth termed PrP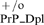, PrP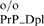, PrP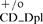 and PrP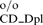 with superscripts defining the *Prnp* allelic status and subscripts denoting the respective hemizygous transgenes).

PrP and Dpl are tethered to the cell membrane by a C-terminal GPI anchor. PrP has been proposed to act as a signal transducer acting on various signaling pathways [9], [10] [27] [28] [29] [[30]], and in this context it was speculated that PrP_ΔCD_ toxicity may require membrane localization. To test this hypothesis, we introduced two point mutations at codons 232 and 233 (original mouse numbering) of the half-genomic construct PrP_ΔCD_ [10], resulting in two in-frame stop codons. This prevents the translation of the carboxy terminal hydrophobic membrane anchoring domain of the precursor protein (see Fig. 1A), resulting in a secreted PrP mutant termed PrP_ΔCDs_. Because of the possible toxicity of the transgene, pronuclear injection was performed into hybrid B6D2F1 *Prnp* ^+/+^ zygotes to generate PrP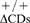 (shorthand as above) transgenic mice. The latter mice were predicted to be viable due to the coexpression of wild-type PrP.

**Figure 2:**
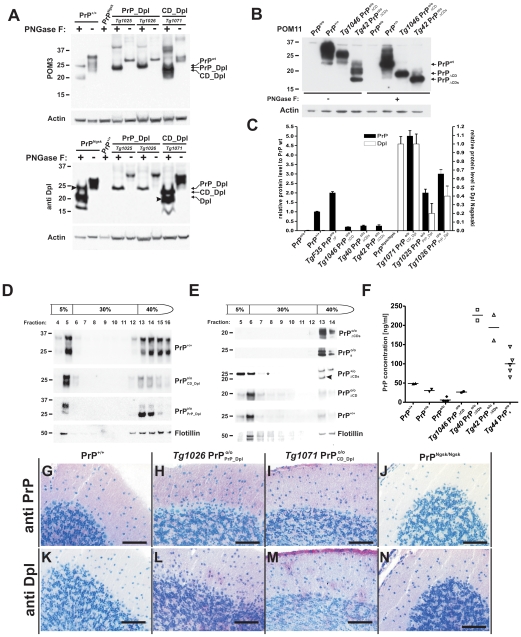
Expression and localization of transgenic proteins. (A) Similar glycosylation patterns of PrP^C^, PrP_Dpl, CD_Dpl and Dpl. Brain homogenates were subjected to PNGase F treatment as indicated, and analyzed by Western blotting using anti-PrP mouse monoclonal antibody POM3 (upper panel) or anti-Dpl mouse monoclonal antibody E2 (lower panel). The spurious band at 20–25 kDa in the 1^st^ lane of the lower panel may indicate incomplete deglycosylation of Dpl. (B) The glycosylation patterns of full-length PrP, PrP_ΔCD_ and PrP_ΔCDs_ are similar. PNGase-treated brain homogenates were analyzed by Western blotting using anti-PrP mouse monoclonal antibody POM11. (C) protein levels in brain extract of transgenic mice compared to PrP in BL/6 mice (filled black columns and left ordinate) and compared Dpl in Nagasaki mice (open columns and right y-axis) using either PrP specific anti bodies POM11 or POM 3 or Dpl specific antibody E2 for western blot. Each column represents the average of 3 mice. (D) Detergent-resistant membrane (DRM) preparations from transgenic mouse brains were separated by density gradient centrifugation and analyzed by Western blotting with monoclonal antibody POM3. Significant amounts of PrP^C^, PrP_Dpl, and CD_Dpl buoyed similarly to flotillin (48 kDa) confirming localization within DRMs. Non-buoyant fractions may indicate raft disruption or may represent immature protein fractions. (E) Density gradient DRM preparations of wild-type and anchorless PrP (PrP_s_), PrP_ΔCD_ and PrP_ΔCDs_ transgenic brains analyzed after deglycosylation with PNGase F with monoclonal antibody POM1. PrP and PrP_ΔCD_ buoyed similarly to flotillin, whereas PrP_s_ and PrP_ΔCDs_ (lower band in fraction 13, arrowhead) were never DRM-associated irrespectively of the presence or absence of wild-type PrP (*). (F) Plasma concentration of prion protein variants. Plasma from wild-type *Prnp* ^+/+^; *Prnp* ^+/o^; *Prnp* ^o/o^; PrP_ΔCD_ (*Tg1046*), PrP_ΔCDs_ (*Tg40; Tg42*) and anchorless PrP_s_ (*Tg44*) mice was studied by ELISA with POM antibodies. PrP plasma levels were vastly elevated in all transgenic mice expressing anchorless versions of PrP. (G–N) Cerebellar sections immunostained with antibodies directed against PrP (POM3) (G–J) and Dpl (K–N). POM3 immunoreactivity was seen in the molecular and granule cell layers of wt (G), PrP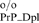 (H) and PrP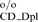 (I) mice but was absent, as expected, from *Prnp* ^Ngsk/Ngsk^ cerebella (J). Cerebellar molecular and granule cell layers are immunostained with anti-Dpl antibody in PrP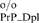 (L) PrP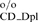 (M) and *Prnp* ^Ngsk/Ngsk^ mice (N). No Dpl staining was observed in wt mice (K) Scale bar 100 µm.

CD_Dpl founder mice #1070, #1071 and #1073, as well as PrP_Dpl founder mice #1023, #1024, #1025 and #1026 and PrP_ΔCDs_ founder mice #36, #37, #38, #39, #40, #41, #42, #43 all exhibited undistorted Mendelian transmission of the transgene when backcrossed to *Prnp* ^o/o^ mice. Transgenic lines were named according to the serial number of their founders. F_2_ generation mice were screened for transgenic integration and expression. One CD_Dpl line (*Tg1071*), two PrP_Dpl lines (*Tg1025* and *Tg1026*) and two PrP_ΔCDs_ lines (*Tg40* and *Tg42*) displayed easily detectable protein expression and were chosen for further analysis (Table 1, Fig. S1, Fig. 2A–C). Quantitative PCR using primers complementary to the common CD sequence (CD_Dpl and PrP_Dpl) or to the 3′-end of the coding region (PrP_ΔCDs_) showed high copy numbers per genome in all transgenic lines, resulting in higher mRNA levels for *Prnp* in wt mice or Dpl in *Prnp* ^Ngsk/Ngsk^ mice (Table 1, Fig. S1).

**Table 1:** Characterization of transgenic mice.

Construct	Deletion	Transgenic copy numbers	mRNA	Protein	Mouse line
PrP^wt^		1	1^+^/0^*^	1^+^	Bl6 WT
PrP_ΔF_	Δ32–134	70	2^+^	2^+^	*TgF35*
PrP_ΔCD_	Δ94–134	1	n.d.	0.2^+^	*Tg1046*
PrP_ΔCDs_	Δ94–134 Δ231–254	6	3.5^+^	0.3^+^	*Tg40*
PrP_ΔCDs_	Δ94–134 Δ231–254	5	3^+^	0.3^+^	*Tg42*
Dpl		1	0^+^/1^*^	1^*^	Bl6 Nagasaki
CD_Dpl		126	1.6^+^/22^*^	5^+^/1^*^	*Tg1071*
PrP_Dpl		180	4^+^/120^*^	2^+^/0.2^*^	*Tg1025*
PrP_Dpl		220	7^+^/180^*^	3^+^/0.4^*^	*Tg1026*

PrP mRNA and protein levels are expressed relatively to wild-type mice (^+^) or, in the case of *Prnp* ^Ngsk/Ngsk^ mice, relatively to Dpl expression (^*^).

Western blots with monoclonal antibody POM3, whose linear epitope was mapped to amino acid residues 95–105 of PrP^C^ [31], revealed significant expression of PrP_Dpl chimeras (2–3 times higher compared to PrP in wt C57BL/6 mice) and of CD_Dpl (5-fold higher than wt C57BL/6 mice; Table 1 and Fig. 2A, C). The expression of CD_Dpl was similar to that of Dpl in *Prnp* ^Ngsk/Ngsk^ mice, whereas PrP_Dpl levels were lower (Fig. 2A, C). The microanatomical distribution of the transgenic proteins resembled that of PrP^C^ (Fig. 2G–N). Western blots of brain homogenates with monoclonal antibody POM11, whose epitope encompasses amino acids 64–72 and 72–80 [10], [31], revealed significant expression of PrP_ΔCDs_ (20–30% of wt C57BL/6 mice, Table 1 and Fig. 2B–C).

The levels of PrP_ΔCDs_ in brains of both transgenic lines *Tg40* and *Tg42* was similar to that of *Tg1046* PrP_ΔCD_ [10] (Fig. 2B, C) and paralleled the measured amount of mRNA (Fig. S1). PrP_ΔCDs_ showed a higher electrophoretic mobility than PrP_ΔCD_ by 2–3 kDa, indicative of the missing GPI anchor.

Upon PNGase-F treatment, the complex banding pattern of PrP_Dpl, CD_Dpl, PrP^C^, PrP_ΔCDs_, and PrP_ΔCD_ was reduced to one single band of lower molecular weight (Fig. 2A–B), suggesting that these proteins were N-glycosylated. The strong reducing conditions prior to PNGase-F treatment prevented recognition of Dpl by anti-Dpl antibody (data not shown). suggesting that this antibody recognizes a discontinuous C-terminal epitope destroyed by reduction of the two disulfide bridges of Dpl. Milder pretreatment resulted in partial deglycosylation of Dpl (Fig. 2A, arrowhead); under these conditions CD_Dpl extracts gave rise to two additional bands, which may indicate posttranslational cleavage (Fig. 2A, arrowhead). PrP_Dpl extracts did not show this phenomenon.

We then prepared detergent-resistant membranes (DRMs) from wild-type, PrP_Dpl and CD_Dpl, (Fig. 2D), PrP_ΔCDs,_ anchorless PrP_s_, and PrP_ΔCD_ brains (Fig. 2E) in the presence or absence of PrP^C^. The buoyancy of PrP_Dpl, CD_Dpl, and PrP_ΔCD_ was similar to that of PrP^C^ and flotillin (Fig. 2D–E), suggesting that they all reside in similar membrane microdomains. Therefore, most aspects of PrP_Dpl and CD_Dpl biogenesis appear to be similar to those of PrP^C^. In contrast, both PrP_ΔCDs_ and PrP_s_ displayed less buoyancy, suggesting no association with rafts in agreement with their biogenesis as soluble proteins. We then prepared DRMs from *Tg42* PrP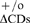 mice coexpressing PrP^C^ and PrP_ΔCDs_. Fractions were deglycosylated with PNGase F prior to western blotting. This experiment revealed that coexpression of wild-type PrP fails to recruit PrP_ΔCDs_ to DRMs. Upon pretreatment with phosphatidylinositol-specific phospholipase C (PI-PLC) the buoyancy of the GPI-anchored PrP variants became similar to that of their anchorless counterparts (Fig. S2).

Finally, we determined the serum PrP concentration in PrP^wt^, PrP_ΔCD_, and PrP_ΔCDs_ mice, as well as in GPI-*Tg44* mice expressing anchorless full-length PrP_s_ [32] (Fig. 2F). Despite similar PrP levels in brain homogenates, mice expressing anchorless versions of PrP (PrP_s_ or PrP_ΔCDs_) displayed up to 4-fold higher serum levels. Therefore, PrP_ΔCDs_ underwent normal maturation and glycosylation but was predominantly secreted, similarly to PrP_s_.

### Phenotypes of mice expressing PrP-Dpl chimeric proteins

**Figure 3:**
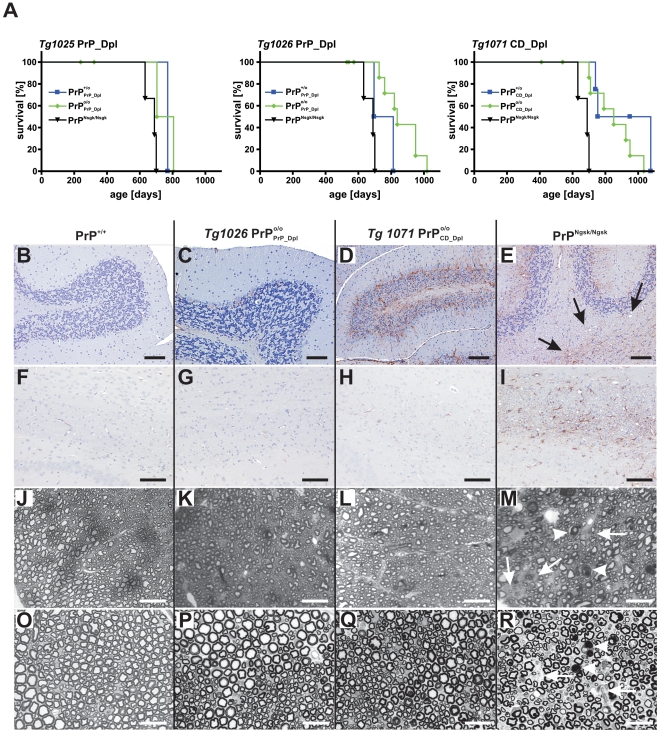
Survival and histological phenotype of PrP/Dpl chimeric mice. (A) Survival of transgenic mice. The longevity of both PrP_Dpl and CD_Dpl mice was unaffected by their endogenous *Prnp* status. All mice survived longer than Nagasaki mice and did not develop clinically apparent pathologies. Each line represents data derived from ≥8 individuals. (B–R) Histopathological changes in 60 week old wt (1^st^ column from left), PrP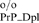 *Tg1026* (2^nd^ column), PrP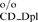 *Tg1071* (3^rd^ column), and PrP^Ngsk/Ngsk^ mice (4^th^ column). Panels B–I represent GFAP immunostains of the cerebellum (1^st^ row) and of the corpus callosum (2^nd^ row), whereas panels J–M depict paraphenylene diamine-stained semithin sections of the mid-thoracic spinal cord (3^rd^ row) and sciatic nerve (4^th^ row). PrP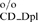 mice showed mild cerebellar astrogliosis (D), whereas PrP^Ngsk/Ngsk^ mice had additional vacuolar white matter changes (arrows) and Purkinje cell loss (E). No pathological changes were seen in *Tg1026* PrP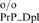 (C), *Tg1025* PrP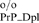 (not shown) and wt mice (B). Vacuolar white matter pathology and astrogliosis in the corpus callosum of PrP^Ngsk/Ngsk^ mice (I) but not in wt (F), PrP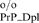 (G) and PrP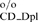 mice (H). Semithin sections revealed coarse vacuolar degeneration of myelinated fiber tracts in PrP^Ngsk/Ngsk^ mice (M, R), whereas no such changes were observed in wt (J, O), PrP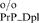 (K, P) and PrP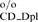 mice (L, Q). Arrows: areas with axonal loss; arrowheads: axons with degenerated myelin sheaths (M, R). Scale bars: 100 µm in panels B–I; 25 µm in panels J–R.

All transgenic lines (*Tg1025; Tg1026; Tg1071*) were maintained in the *Prnp* ^+/o^ or *Prnp* ^o/o^ allelotype (PrP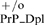, PrP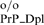, PrP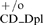 and PrP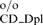), and monitored using a four-degree clinical score [10]. It has previously been shown that onset and development of disease correlate with expression levels of Dpl. Tg(Dpl)28272/ZrchI and (TgN-Dpl)32 mice, which express high amounts of Dpl, survived only 32 and 60 days respectively [18], [19] whereas mice expressing lower Dpl levels, such as *Prnp* ^Ngsk/Ngsk^ mice [16], showed progressive symptoms of ataxia and were euthanized according to clinical scoring at ≥70 weeks of age. Instead, none of the PrP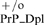, PrP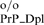, PrP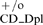 and PrP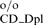 mice showed abnormal behavior even at >100 weeks of age, and most of them died at 26–35 months of age (Fig. 3A). This suggests that the presence of amino terminal domains of PrP reduces the toxicity of Dpl.

### Phenotypes of mice expressing anchorless PrP_ΔCDs_ proteins

**Figure 4:**
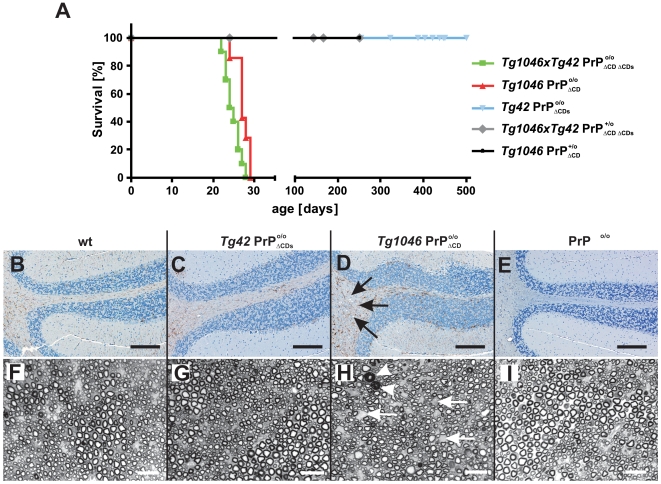
Survival and histological phenotype of transgenic mice expressing PrP_ΔCDs._ (A) Survival of compound transgenic mice. Survival curves of mice expressing *Tg1046* PrP_ΔCD_, *Tg42* PrP_ΔCDs_ or PrP_ΔCD_ and PrP_ΔCDs_ (*Tg1046×Tg42*), in the presence or absence of full-length PrP^C^. Each line comprises the number of individuals indicated in Table 2. (B–I) Histopathological changes in 23 days old wt (B, F), *Tg40* PrP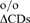, (C, G), and terminal *Tg1046* PrP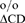 mice (D, H) and *Prnp* ^o/o^ mice (E, I); B–E are GFAP immunostains of cerebellum, F–I are transverse semithin sections of sciatic nerves. Severe astrogliosis and vacuolar changes (arrows) are observed in cerebellar white matter of *Tg1046* PrP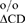 mice (D) as described [10]. No pathological changes are seen in *Tg40* PrP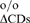 (C) wt (B) and *Prnp* ^o/o^ mice (E). Transverse semithin sections of the sciatic nerve (F–I) reveal mild axonal loss (arrows) and coarse vacuolar degeneration in myelinated fiber tracts in *Tg1046* PrP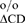 mice (H) while no such changes are observed in wt (F), *Tg40* PrP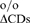 (G) and *Prnp* ^o/o^ mice (I). White arrowheads: axons with degenerated myelin sheaths. Scale bars: 200 µm (B–E) or 20 µm (F–I).

Transgenic lines *Tg40* and *Tg42*, henceforth termed PrP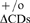, and PrP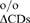, were monitored using the same clinical score as with Dpl-PrP chimeric mice. Onset and development of disease caused by PrP_ΔCD_ correlated inversely with expression levels of the transgene and was ameliorated by coexpression of PrP^C^. Mice expressing high amounts of PrP_ΔCD_ survived 35 (*Tg1050*) or 80 days (*Tg1047*) in a PrP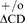 genotype whereas *Tg1046 mice*, which express less PrP_ΔCD_ only developed pathology in the absence of PrP^C^ and reached an age of 26 days [10]. Despite higher total expression levels in PrP_ΔCDs_ than in PrP_ΔCD_, even after >60 weeks none of the PrP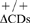, PrP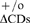, and PrP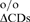 from both transgenic lines *Tg40* and *Tg42* showed abnormal behavior, and most of them died at a similarly advanced age as wt mice (Fig. 4A). Therefore, removal of the membrane anchor prevents the toxicity caused by deletion of the central domain (CD) of PrP^C^.

### Histological phenotype

Wt, PrP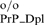, PrP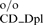, and *Prnp* ^Ngsk/Ngsk^ mice were sacrificed at 100, 200, and 420 days of age, and brains as well as spinal cords were analyzed histologically. By the age of 200 days these mice displayed no pathological alterations with the exception of some Purkinje cells loss in *Prnp* ^Ngsk/Ngsk^ mice (data not shown). When brains of 60 week-old wt (Fig. 3B, F, J), *Tg1026* PrP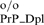 (Fig. 3C, G, K), *Tg1071* PrP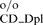 (Fig. 3D, H, L) and *Prnp* ^Ngsk/Ngsk^ mice (Fig. 3E, I, M) were compared, GFAP immunostains (Fig. 3B–I) showed moderate activation of astrocytes within the molecular layer of the cerebellum in PrP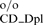 mice (Fig. 3D). No such pathological changes were seen in PrP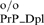 (Fig. 3C) and wt mice (Fig. 3B).

White matter pathology characterized by vacuolation and astrogliosis was seen in the cerebellum (arrows Fig. 3E) and in the corpus callosum of *Prnp* ^Ngsk/Ngsk^ mice (Fig. 3I). None of these changes were observed in wt, *Tg1026* PrP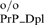 and *Tg1071* PrP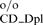 mice (Fig. 3F–H). Transverse semithin sections of spinal cords (mid-thoracic level, Fig. 3J–M) and of sciatic nerves (Fig. 3O–R) revealed coarse vacuolar degeneration (white arrowheads) in myelinated fiber tracts in *Prnp* ^Ngsk/Ngsk^ mice and axonal loss (white arrows, Fig. 3M, R). No such changes were observed in wt, *Tg1026* PrP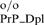 and *Tg1071* PrP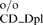 mice (Fig. 3J–L, O–Q).

Wt, *Tg1046* PrP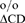, *Tg40* PrP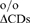, and *Prnp* ^o/o^ mice were sacrificed at 23 days and 60 weeks of age, and brains as well as sciatic nerves were analyzed histologically (Fig. 4). *Tg40* PrP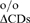 mice of 23 days of age (Fig. 4C, G) displayed no pathological alterations compared to *Tg1046* PrP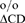 mice which showed strong cerebellar white-matter astrogliosis (Fig. 4D). Transverse semithin sections of the sciatic nerve revealed peripheral neuropathy in *Tg1046* PrP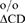 with axonal loss white arrows and myelin degeneration white arrowheads (Fig. 4H) but not in wt (Fig. 4F), PrP^o/o^ (Fig. 4I) or *Tg40* PrP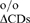 (Fig. 4G) at 23 days of age. No PrP_ΔCDs_ toxicity was observed also at later time points (data not shown).

### Functional rescue of truncated PrP variants

**Figure 5:**
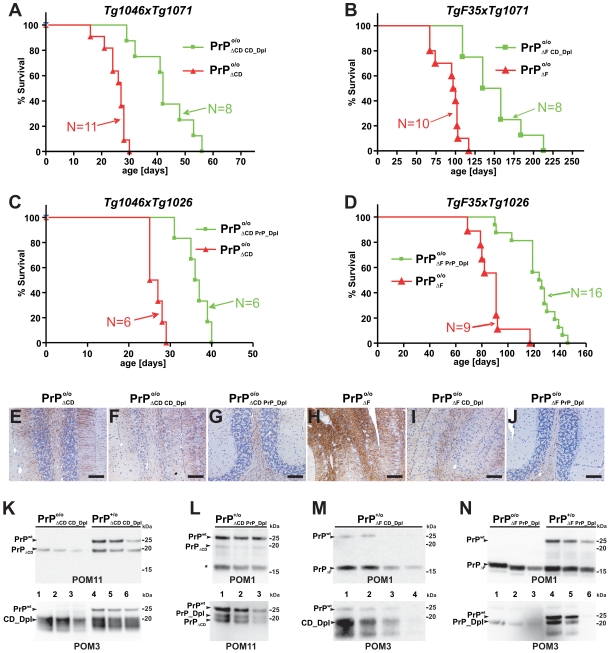
Survival of compound transgenic mice. Survival of compound transgenic mice derived from intercrosses between the transgenic lines described above. (A–D) Survival curves of mice lacking PrP^C^ and expressing various transgenes (PrP_ΔF_, PrP_ΔCD_, CD_Dpl, PrP_Dpl) as indicated by the subscripts. Each line summarizes the survival animals with the respective genotype (group size: 6–16 as indicated). (E–J) Comparison of histopathological phenotypes in terminally sick PrP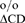 and PrP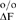 mice with their respective age matched compound littermate transgenic mice. All pictures show GFAP-immunostained cerebellar sections at identical magnification. Terminally sick *Tg1046* PrP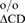 mice showed astrogliosis both in cerebellar cortex and white matter (E). Milder changes were present in *Tg1046×Tg1071* PrP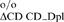 (F) and *Tg1046×Tg1026* PrP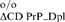 mice (G). Subtotal granule cell loss associated with severe astrogliosis was seen in the cerebellum of *TgF35* PrP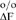 mice (H). However, granule cell loss and astrogliosis was less severe in *TgF35×Tg1071* PrP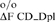 mice (I) and almost absent from *TgF35×Tg1026* PrP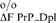 mice (J). Scale bar = 5 µm. (K–N) Brain expression of PrP^C^ and transgenic PrP deletion mutant as well as PrP/Dpl fusion proteins. Specific bands are indicated with arrowheads (K) The expression of CD_Dpl was higher than that of PrP_ΔCD_ and PrP^C^. Lanes 1–3 represent a serial dilution of a *Tg1046×Tg1071* PrP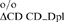 mouse compared to a PrP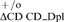 mouse (lanes 4–6). (L) PrP_Dpl expression is similar to that of PrP_ΔCD_ and significantly lower than that of PrP^C^. Lanes 1–3 represent serial dilutions of *Tg1046×Tg1026* PrP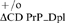 mice. The asterisk indicates a carboxy terminal fragment formed from wild-type PrP^C^. (M) Indirect comparison indicates similar PrP_ΔF_ and CD_Dpl levels in *TgF35×Tg1071* PrP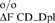 mice which were higher than those of PrP^C^. Lanes 1–4 depict a serial dilution of a *TgF35×Tg1071* PrP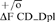 mouse. (N) PrP_Dpl was less abundant than PrP_ΔF_ in *TgF35×Tg1026* PrP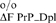 and *TgF35×Tg1026* PrP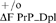 mice. Lanes 1–3 depict a serial dilution of a *TgF35×Tg1026* PrP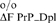 mouse compared to a *TgF35×Tg1026* PrP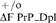 mouse (lanes 4–6). All brain homogenates were treated with PNGase F, and replica western blots were decorated with antibodies POM1, POM3, and POM11 as indicated below each blot.

PrP_Dpl and CD_Dpl did not elicit any clinical or histopathological syndrome in *Prnp* ^o/o^ mice. This may indicate that PrP_Dpl and CD_Dpl have lost all functional characteristics of PrP-like proteins. We assessed this possibility by intercrossing *Tg1026* PrP_Dpl and *Tg1071* CD_Dpl transgenic mice with the neurotoxic PrP deletion mutants *Tg1046* PrP_ΔCD_ and *TgF35* PrP_ΔF_ mice [9], whose toxicity can be ameliorated by the coexpression of full length PrP. The resulting *Tg1046* PrP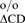 developed first signs of disease at 18–20 days post birth and reached terminal disease at 25±0.7 days (n = 22) of age, as described previously. Double transgenic *Tg1046×Tg1071* PrP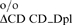 mice survived until 43±3.3 days (n = 8), whereas *Tg1046×Tg1071* PrP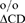 littermates survived 25±2.0 days (n = 11) (Fig. 5A and Table 2). Double-transgenic *Tg1046×Tg1026* PrP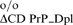 mice survived 36±1.3 days (n = 6), as opposed to 26±1.7 days (n = 6) for *Tg1046×Tg1026* PrP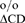 littermates (Fig. 5C and Table 2). A similar trend was also seen in the transgenic line *Tg1025* PrP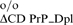 and in intercrosses of the PrP_ΔCD_ lines *Tg1047* and *Tg1050* (data not shown), with significant prolongation of survival (ANOVA; *p*<0.001). *TgF35* PrP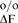 mice developed ataxia and were euthanized at 96±5.3 days of age (Fig. 5B, D and Table 2) as described [9]. Double transgenic *TgF35×Tg1071* PrP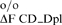 mice survived 150±12.7 days (n = 8; Fig. 5B; Table 2), whereas double transgenic *TgF35×Tg1026* PrP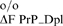 mice survived 139±4.13 days (n = 11; Fig. 5D; Table 2). In both cases survival was significantly longer (ANOVA; *p*<0.001) than for the single transgenic littermates *TgF35×Tg1071* PrP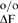 and *TgF35×Tg1026* PrP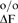. In both paradigms one *Prnp* allele sufficed to fully suppress the phenotype of the toxic mutant (data not shown).

**Table 2:** Survival of compound transgenic mice.

Crosses	Number of animals	Genotype	Average survival [days]	Standard deviation of mean [days]	Significance
*Tg1046*	22	PrP 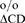	25.2	0.7	
*Tg1046×Tg1071*	8	PrP 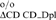	42.9	3.3	*** p<0.001
*Tg1046×Tg1026*	6	PrP 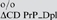	36.3	1.3	*** p<0.001
*TgF35*	15	PrP 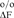	95.6	5.3	
*TgF35×Tg1071*	8	PrP 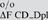	150.1	12.7	*** p<0.001
*TgF35×Tg1026*	11	PrP 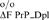	139.1	4.1	*** p<0.001
*Tg1046*	7	PrP 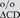	27.3	0.6	
*Tg1046×Tg42*	10	PrP 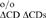	24.8	0.6	Ns p>0.05

Mice of various genotypes were housed and monitored according to a 4-degree clinical score system. Terminally sick animals were euthanized. Mean survivals of single-transgenic littermates were compared to double transgenic mice and statistical significance of difference was tested by ANOVA.

Histological analysis of terminally sick *Tg1046* PrP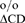 mouse brains revealed astrogliosis both in the corpus callosum (not shown) and in the cerebellar white matter (Fig. 5E) while *TgF35* PrP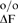 mice displayed additional severe cerebellar granule cell (CGC) loss (Fig. 5H). Milder white-matter changes and much less severe CGC loss were observed in compound *Tg1046×Tg1071* PrP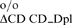 and *Tg1046×Tg1026* PrP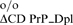 littermates euthanized at the same age (Fig. 5F–G and 5I–J). Western blot analysis of brain homogenates indicated that expression levels of the various transgenic proteins were unchanged in the compound transgenic mice independently of the respective combination. The steady-state levels of CD_Dpl exceeded those of PrP_ΔCD_ PrP_ΔF_ and PrP^wt^ (Fig. 5K, M), whereas those of PrP_Dpl and PrP_ΔCD_ were similar and much lower than those of PrP_ΔF_ (Fig. 5L, N). Although expression of CD_Dpl was higher than that of PrP_Dpl, and compound PrP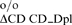 and PrP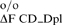 mice displayed longer survival than PrP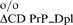 and PrP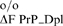 mice, CD_Dpl seemed to be less effective than PrP_Dpl to suppress cerebellar granule cell loss. This finding may point to a specific function of the amino proximal regions in suppressing neurodegeneration.

In order to address the functionality of PrP_ΔCDs_, we intercrossed *Tg42* PrP_ΔCDs_ and *Tg1046* PrP_ΔCD_ mice and monitored the offspring for clinical signs of disease. *Tg1046*×*Tg42* PrP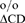 were found to develop first signs of disease at 18–20 days post birth, and reached terminal disease at 25±0.71 days of age (n = 22; Fig. 4A and Table 2). Double transgenic *Tg1046×Tg42* PrP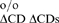 mice survived for 25±1.9 days. Hence there was no significant difference in survival. All single or double transgenic mice coexpressing PrP^C^: *Tg1046×Tg42* PrP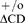, and *Tg1046×Tg42* PrP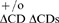 survived to old age without any signs of clinical disease, indicating that PrP_ΔCDs_ does not diminish the potential of PrP^C^ to ameliorate PrP_ΔCD_ induced toxicity. In contrast, PrP_ΔF_ and PrP_ΔCD_ were previously shown to compete for the rescue effect of PrP^C^ in double transgenic mice *Tg1046×TgF35* PrP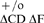 [10]. We therefore conclude that removal of the lipid anchor from PrP_ΔCD_ completely abolishes its neurotoxic properties.

## Discussion

The results presented here confirm and extend a recent report that fusion of the complete amino-terminus of PrP detoxifies Dpl. Tg(PrPN-Dpl) mice expressing a fusion protein consisting of amino acids 1–124 of PrP and amino acids 58–179 of Dpl failed to show Dpl typical neurological disorder and were able to prolong the onset of ataxia in mice with exogenous Dpl expression [24]. By generating chimeric proteins that contain either the entire amino-terminus of PrP linked to the carboxy-terminus of Dpl (PrP_Dpl) or the central domain of PrP alone (CD_Dpl), we found specific domains within the amino-terminus of PrP that are involved in the detoxification of Dpl in two distinct brain regions and cell types.

While PrP_Dpl showed no signs of cerebellar granule cell degeneration for at least 60 weeks, PrP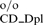 mice displayed mild astrogliosis within the CGC layer. This may point to some residual neurotoxicity of CD_Dpl. In contrast, white matter degeneration was observed in Dpl-expressing Ngsk mice yet was not seen in mice expressing either of the two transgenes, PrP_Dpl and CD_Dpl. Since leukoencephalopathy is the major life-shortening pathology associated with expression of truncated PrP and Dpl [10], [33], both addition of the whole amino-terminus, or addition of the central domain alone resulted in a normal life expectancy in transgenic mice.

In addition to detoxifying Dpl, chimeric fusion proteins were able to partially antagonize the toxic effects of the PrP deletion mutants PrP_ΔF_ and PrP_ΔCD_. While PrP_Dpl was able to antagonize cerebellar granule cell loss in PrP_ΔF_ mice, CD_Dpl was not. Cerebellar white matter gliosis was milder in both PrP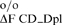 and PrP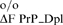 mice. This lends further support to the conclusion that distinct domains within PrP exert neurotrophic functions in a variety of brain regions and cell types.

We have excluded that differences in expression level were responsible for the observed effects: Western blotting with antibody POM3 [31], which recognizes a domain common to both transgenes, showed a higher expression for CD_Dpl than for PrP_Dpl. All transgenic constructs were expressed using the same backbone, thereby reducing the likelihood of differential expression in distinct cell types. Thus the cell-specific effects of the different transgenes appear to be related to their structural features rather than to the levels or tissue-specific patterns of their expression.

Despite sequence homologies of <20%, the carboxy terminal domains of Dpl and PrP have very similar folding patterns of the respective carboxy proximal regions, whereas their amino proximal portions are much less structured [20], [34], [35]. Hence the selective permutations of the less structured domains of the two proteins performed here are not very likely to alter the overall global fold of the resulting fusion proteins. We found that both PrP_Dpl and CD_Dpl underwent correct intracellular sorting and posttranslational processing (Fig. 2A, C). Furthermore, in none of the transgenic mice (including the lines expressing the highest levels of transgene) did we detect any spontaneous formation of PK-resistant transgenic protein or PrP aggregates by Western blotting and histology (data not shown).

Further evidence for specific differences in the function of PrP^C^ comes from the previous studies on transgenic mice expressing PrP^C^ in a cell-type specific manner. While cerebellar granule cell loss in PrP_ΔF_ mice was reversed by neuronal expression of PrP, white matter degeneration was rescued by myelin-specific expression of PrP [36].

Cell-specific requirements for distinct PrP domains might explain the discrepancies regarding the domains reported to be involved in cytotrophic functions. Several studies suggest that the octapeptide repeat region is crucially linked to the neuroprotective functions of PrP^C^ [37], [38], [39]. On the other hand, a feature common to all the toxic PrP deletion mutants is the lack of the central domain (encompassing at least residues 105–125) within PrP^C^. This in turn points to a role of the central domain of PrP^C^.

The results presented here may help clarifying this controversy. The central domain (aa 94–134) appears to be crucial for myelin maintenance, while other domains within the amino terminus (aa 23–94) may be required for neuroprotection. Residues 23–94 consists of the amino-terminal charged cluster (aa 23–28) involved in endocytosis and of the octapeptide repeat region associated with neuroprotection via anti-oxidative function and copper binding (aa 50–90) [37], [39], [40]. It was initially reported that amino acids 23–88 are needed to fully suppress neurotoxicity on Purkinje cells [41], yet it was later shown that the octapeptide repeats are dispensable for this function. This suggests that the charge cluster may be more relevevant for the neuroprotection of Purkinje cells [24] that for other cell types. It is less likely that toxic domains within the amino-terminus of Dpl in CD_Dpl may be responsible for the observed residual neurotoxicity, since earlier studies showed that the proximate cause of cerebellar granule cell degeneration is not the amino terminus of Dpl, but rather its carboxy terminus [42].

PrP^C^ was reported to inhibit the NR2D subunits of the NMDA receptor complex, and *Prnp^o/o^* hippocampal neurons display increased neuronal excitability and enhanced glutamate excitotoxicity [43]. It will be interesting to study whether chimeric PrP/Dpl proteins exert PrP^C^ like functional regulation of the NMDA receptor and whether central domain, octapeptide repeat region or amino-terminal charged cluster are involved in this function.

It was suggested that homodimerization of PrP^C^ mediates the transduction of extracellular signals [44], [45], [46]. The toxicity of truncated PrP and Dpl is counteracted by overexpression of full-length PrP^C^ [9], [10], [18], [19] and exacerbated by removal of the endogenous *Prnp* gene, suggesting that PrP^C^ and its variants compete for a common interacting molecule. The PrP/Dpl fusion proteins appear to partake in this competition as well, as both CD_Dpl and PrP_Dpl prolonged survival of PrP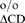 and PrP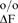 mice. Perhaps the CD region is responsible for stringent protein-protein interactions, whereas the structured carboxy termini of PrP and Dpl allow for more relaxed interactions and are therefore interchangeable. Such interactions might also include the formation of functionally relevant homodimers or homooligomers [47]. The residues 113–128 of PrP mediate interaction of PrP with stress inducible protein 1 (STI) [48] and heparan sulfate [49]. The incompleteness of the rescue in all tested paradigms of PrP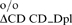, PrP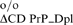, PrP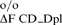 and PrP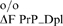 mice may relate to insufficient amounts of the respective fusion proteins, or possibly to reduced affinity for their binding partners.

In addition to the findings described above, we extended our analysis of functional domains within PrP to those determining the localization of the protein. Mice expressing anchorless PrP accumulate high titers of prions and protease-resistant PrP when challenged with scrapie [32], [50], yet develop only subtle pathologies [51]. Here, anchorless PrP_ΔCDs_ was expressed to high levels in transgenic mice, and was very efficiently secreted into the extracellular space of brain and in serum as a mature, fully glycosylated soluble form [52]. Although the deletion within PrP_ΔCDs_ was identical to that of the neurotoxic membrane anchored PrP_ΔCD_, it did not induce any pathology in transgenic mice, irrespectively of the presence or absence of full-length PrP^C^. Since the total concentration of PrP_ΔCDs_ in brain homogenates was as high as that of PrP_ΔCD_, and even higher than that of PrP_ΔCD_ in the serum, lack of toxicity was unrelated to its expression level. Also, PrP_ΔCDs_ failed to influence the survival of PrP_ΔCD_ mice coexpressing PrP^C^, confirming that it exerts neither beneficial nor detrimental effects on the central nervous system.

PrP_ΔCDs_ did not localize to detergent-resistant membrane (DRM) fractions, even when wild-type PrP^C^ was coexpressed. This observation suggests that the genetic interaction between PrP^C^ and its neurotoxic variants may physically necessitate membrane anchoring of all relevant partners. In contrast, soluble-dimeric prion protein (PrP-Fc_2_) was found to translocate to the DRM compartment and to associate with PrP^Sc^ upon prion infection of mice coexpressing PrP^C^ and PrP-Fc_2_ [51]. In this context, it may be of interest to study the localization of PrP_ΔCDs_ in prion infected mice.

In conclusion, the above findings indicate that (1) the amino proximal domain of PrP contains minimal elements that are necessary and sufficient for PrP function, that (2) distinct domains within the amino-terminus of PrP exert site- and/or cell-specific functions, and that (3) GPI membrane anchoring is mandatory for exerting said function. The understanding of the physiological and pathophysiological functions of the prion protein will benefit from functional analyses of the proteinaceous [48] and non proteinaceous [49] constituents interacting with PrP and its variants. Finally, it will be of particular interest to explore whether the phenomena studied here share functional and molecular aspects with the neurotoxicity observed in prion diseases [53].

## Materials and Methods

### Ethics Statement

All mice were maintained under specific pathogen-free (SPF) conditions. Housing and experimental protocols were in accordance with the Swiss Animal Protection Law and in compliance with the regulations of the Veterinaeramt, Kanton Zurich.

### Construction of the transgenes

The coding region of murine *Prnp* and Prnd gene were analyzed using DNAMAN software (Lynnon BioSoft, Canada), and hydrophobicity plots were generated using a window of 9 amino acid residues. The regions identified in these plots were used to define the CC, CD and HC domains. The chimeric fusion proteins of PrP and Dpl were designed such that their hydrophobicity characteristics would mimic that of wild-type PrP. Based on pPrPHG [25], a *Pme*I/*Nhe*I fragment was subcloned in the pMECA [54] backbone. To create the CD_Dpl cDNA, mouse genomic cDNA was used as template to obtain two PCR fragments with primer sets JP1 (5′-ATA ATA ATG CAT ACC ACC ATG AAG AAC CGG CTG GGT AC)/JP2 (5′-TAC TGC CCC AGC TGC CGC AGC CCC TGC CAC ATG CTT GAG GTT GGT TTT TGG TTT GCT GGG CTT GTT CCA CTG ATT ATG GGT ACC CCC TCC CCG GCC TTG CTT GAT GAA GG) and JP3 (5′-CCT CAA GCA TGT GGC AGG GGC TGC GGC AGC TGG GGC AGT AGT GGG GGG CCT TGG TGG CTA CAT GCT GGG GAG CGC CGT GAG CAG GCC CAT GAA GCT GGA CAT CGACTT TGG )/JP4 (5′-ATA ATA ATG CAT TTA CTT CAC AAT GAA CCA AAC). The two initial products were fused in a third PCR with the flanking primers JP1 and JP4. This product was digested with *Nsi*I and ligated to the *Nsi*I sites of the pMECA vector containing the pPrPHG subcloned *Pme*I/*Nhe*I sequence into which a second *Nsi*I site had been engineered. After confirming insertion with the correct orientation, the insert was cloned back into the pPrPHG backbone using the *Pme*I/*Nhe*I sites.

PrP_Dpl was created based on the plasmid pPrPHG [25]. A fragment (480 bp) was amplified using the primers pE2* (5′-CAA CCG AGC TGA AGC ATT CTG CCT)/X2 (5′-CCT GCT CAC GGC GCT CCC CAG CAT G) containing sequence information from Exon3 to codon 132/133 of the murine PrP. In a second PCR using genomic DNA as template and primers X3 (5′-GGG AGC GCC GAC ATC GAC)/X4 (5′-AAA GAA TTC CAC AAT TCT TAC TTC ACA ATG) a fragment (360 bp) containing codon 68 until polyadenylation site of Dpl was amplified. After purification both fragments were cut with *Hae*II mixed and directly ligated into the pCR-Blunt II-Topo vector. The transgene was then excised with *Age*I/*Eco*RI and, after blunting the 3′ *Eco*RI sites, ligated into the original *Age*I/BbrPI site of pPrPHG. The presence of the new insert was confirmed by restriction analysis using *Sma*I.

PrP_ΔCDs_ was generated using the pMECA *Pme*I/*Nhe*I subclone pPrPHG previously described [10]. The oligonucleotide primers dCDSol5′ (5′-CCT ATT ACG ACG GGA GAA GAT CCT GAT GAA CCG TGC TTT TCT CCT CC-3′) dCDSol3′ (5′- GGA GGA GAA AAG CAC GGT TCA TCA GGA TCT TCT CCC GTC GTA ATA GG-3′), each complementary to opposite strands of the vector, were extended during temperature cycling by *PfuTurbo*® DNA polymerase. On incorporation of the oligonucleotide primers, a mutated plasmid containing staggered nicks was generated. After temperature cycling and treatment with *Dpn*I to digest the parental DNA template and select for the desired DNA construct, the nicked vector DNA incorporating the mutations was transformed into E. coli. Clones were picked and sequenced. Finally the *Pme*I/*Nhe*I fragment containing the desired point mutation was religated into the pPrPHG vector as described before [10].

### Generation, Identification, and Maintenance of Transgenic Mice

The pPrPHG plasmids containing the PrP or Dpl coding sequences were propagated in E. coli XL1 blue, the minigene excised with *Not*I and *Sal*I, and processed as described [25]. Pronuclear injections into fertilized oocytes were carried out as described [55]. Transgenes on a *Prnp* ^o/o^ background were identified by PCR using the exon 2 primer pE2* (5′-CAA CCG AGC TGA AGC ATT CTG CCT) and the exon 3 primer Ubl floxed Dpl (5′-CTC GCT GGT GGA GCT TGC TAT C) resulting in a PCR product of 618 bp for CD_Dpl and 670 bp for PrP_Dpl or pE2* and exon 3 primer Mut217 (5′-CCT GGG ACT CCT TCT GGT ACC GGG TGA CGC) resulting in a PCR product of 619 bp. PCR analysis in order to verify the outbreeding of the *Prnp* ^+^ allele was carried out using primers P10 (*Prnp* exon 3, 5′-GTA CCC ATA ATC AGT GGA ACA AGC CCA GC), 3′NC (non-coding region at 3′ of exon 3, 5′-CCC TCC CCC AGC CTA GAC CAC GA), and P3 (neoR gene, 5′-ATT CGC AGC GCA TCG CCT TCT ATC GCC); P10 and 3′NC gave an 560 bp signal for the *Prnp* ^+^ allele, and P3 and 3′NC gave a 362 bp product for the *Prnp* ^0^ allele. Alternatively, to test for the presence or absence of the *Prnp* ^+^ allele an additional PCR was performed using primers P2 (*Prnp* int 2, 5′-ATA CTG GGC ACT GAT ACC TTG TTC CTC AT) and P10rev (reverse complementary of P10 5′-GCT GGG CTT GTT CCA CTG ATT ATG GGT AC) giving a product of 352 bp for the *Prnp* ^+^ allele. In order to distinguish between transgenic mice expressing PrP_ΔCD_ and PrP_ΔCDs_, two separate PCR reactions were performed using primers pE2* and pdCDrev (5′-GGA GGA GAA AAG CAC GGT GCT GCT) yielding a diagnostic amplicon of 666 bp, or using pE2* and pdCDsrev (5′-GGA GGA GAA AAG CAC GGT TCA TCA) yielding a diagnostic amplicon of 666 bp.

### Q-PCR to determine genomic copy numbers

Total genomic DNA was prepared from mouse tails after PK digestion and purified according to standard procedures. Copy numbers were assessed by Taqman PCR using 2 ng of total genomic DNA and primer pairs CD Sonde5′ (5′-GGA GGG GGT ACC CAT AAT) and CD Sonde3′ (5′- GCG CTC CCC AGC ATG TAG) on C57Bl6, *Tga20*, *Prnp* ^o/o^, *Tg1025*, Tg1026 and *Tg1071* mice. For determination of copy numbers of *Tg40*, *Tg42* primer pairs p60 (5′-CGC TAC CCT AAC CAA GTG T) and p61 (5′-GAT CTT CTC CCG TCG TAA T) were used. To standardize Taqman PCR on GAPDH using primers GAPDH up (5′-CCA CCC CAG CAA GGA GAC T) and GAPDH down (5′-GAA ATT GTG AGG GAG ATG CT) was done in parallel.

### mRNA analysis

Total brain RNA was isolated in Trizol (Life Technologies), purified and DNase treated according to the manufacturer's manual (Roche). After reverse transcription (Geneamp; Roche) cDNA was used for Taqman PCR using primer pairs Dpl Taq5′ (5′-CTA CGC GGC TAA CTA TTG)/Dpl Taq3′ (5′-CGC CGG TTG GTC CAC) and PrP Taq5′ (5′-CAG TGG AAC AAG CCC AGC)/PrP Taq3′ (5′-CCC CAG CAT GTA GCC ACC). To standardize expression levels GAPDH using primers GAPDH up (5′-CCA CCC CAG CAA GGA GAC T) and GAPDH down (5′-GAA ATT GTG AGG GAG ATG CT) and 18S rRNA using primers 18S fw (5′-GTA ACC CGT TGA ACC CCA TT) and 18S rc (5′-CCA TCC AAT CGG TAG TAG CG) were used. Taqman PCR using SYBR-green (Roche) and determination of ΔΔCT-values were done on a Applied Biosystems 7900 device. As control for possible DNA contamination, DNase-treated RNA from wt and tg mice that had not been reversely transcribed was used.

### Western blot analysis

Brain hemispheres were homogenized in 7 vol PBS, 0.5% Nonidet P-40, and 0.5% deoxycholate and the solution was centrifuged 5 min in an Eppendorf centrifuge. For deglycosylation, up to 50 µg denatured total protein were incubated at 37°C for 4 h with 500 U PNGase F (New England Biolabs) according to the manufacturer's instructions. The protease inhibitors Pefabloc (1 mg/ml), Leupeptin (10 µg/ml), Pepstatin (10 µg/ml), Aprotinin (1 µg/ml) (all from Boehringer, Mannheim), and 0.5 mg/ml EDTA were added. After electrophoresis of protein samples through 12% SDS-polyacrylamide gels, samples were transferred to nitrocellulose membranes (Schleicher & Schuell) and incubated with mouse monoclonal anti-PrP antibodies POM1, POM3 and POM11 [31], followed by incubation with peroxidase-labeled anti-mouse antiserum (1∶2500; Amersham) and developed with the ECL detection system (Pierce). Antibody incubations were performed in 1% Top Block (Juro) in Tris-buffered saline-Tween (TBS-T) for 1 h at room temperature or overnight at 4°C.

### Flotation assays

Flotation of detergent insoluble complexes was performed as described [56]. Appropriate brain homogenates were extracted for 2 h on ice in cold lysis buffer (150 mM NaCl, 25 mM Tris-HCl, pH 7.5, 5 mM EDTA, 1% Triton X-100; total protein: 1 mg in 1.6 ml. Extracts were mixed with two volumes (3.2 ml) of 60% Optiprep® (Nycomed) to reach a final concentration of 40%. All lysates were loaded at the bottom of Beckman ultracentrifuge tubes. A 5–30% Optiprep® step gradient in TNE (150 mM NaCl, 25 mM Tris-HCl, pH 7.5, 5 mM EDTA) was then overlaid onto the lysate (8.4 ml of 30% Optiprep® and 3.6 ml of 5% Optiprep®). Tubes were centrifuged for 24 h at 4°C in a TLS55 Beckman rotor at 100,000 *g*. Fractions (1 ml) were collected from the top of the tube and processed for immunoblotting and visualization with anti-PrP antibody POM3 [31], anti-flotillin 1, and anti-GAPDH antibody (both BD Transduction Laboratories). In order to release GPI anchored proteins from membranes, brain homogenates were treated for 2 h at 37°C with 10 U/ml Phospholipase C (PI-PLC from Sigma) as described [57].

### ELISA

PrP ELISA was performed as described in [58] 96-well plates (Nunc-Immuno Maxisorb; prod. no. 439454) were coated with 50 µL per well of POM1 (2 mg/ml, 1∶5000 in 0.1 M sodium carbonate buffer pH 9.6 [1.58 g Na_2_CO_3_+2.94 g NaHCO_3_ in 500 ml H_2_O]) over night at 4°C. All following incubation steps were made at room temperature. The plates were washed by immersing them 4–5 times in PBS with 0.1% Tween-20 (PBST). Plates were then incubated with 100 µL per well of blocking buffer (5% Top-Block in PBST) for two hours. A 1∶3 dilution of recombinant murine PrP (rmPrP) (starting from 50 ng/ml) was used for a standard curve. Blood plasma from respective mice was diluted appropriately in sample buffer (1% Top-Block in PBST) and incubated for 1 h. Then, plates were washed 4–5 times in PBST and incubated with biotin-labeled POM2 (1 mg/ml, 1∶5000 in sample buffer, 100 µL per well) for 1 h. Plates were washed 4–5 times and incubated with avidin-HRP (1 mg/ml, 1∶1000 in sample buffer, 100 µL per well) for 1 h followed by another round of washing, 4–5 times in PBST and 2–3 times with PBS alone. Chromogenic substrate (Biosource, prod. no. SB02, 50 µL per well) was applied for up to 10 min. The reaction was stopped with 0.5 M H_2_SO_4_ and absorbance was read at 450 nm.

### Clinical scoring and observation

Mice were examined once weekly for clinical signs as described previously [10]. Mice were euthanized when they reached a score of 3.5 or higher. Statistical significance was assessed as indicated.

### Morphological analyses

Brains, spinal cords and sciatic nerves were removed and fixed in 4% formaldehyde in PBS, pH 7.5, paraffin embedded, and cut into 2–4 µm sections. Sections were stained with hematoxylin-eosin (H&E), Luxol-Nissl (myelin and neurons), and commercial antibodies to GFAP (glial fibrillary acidic protein; activated astrocytes), MBP (myelin basic protein), NF200 (neurofilament 200), IBA1 (microglia) and SAF84 (PrP^Sc^ aggregates). For semithin sections and electron microscopy mice were perfused with ice-cold 4% PFA/3.9% glutaraldehyde. Spinal cord tissues were removed, immersed in the same solutions, and kept in Phosphate buffer at 4°C until processing. Tissues were embedded in Epon, and semithin sections were stained with toluidine blue and paraphenylene diamine. Frozen sections for POM3 and Dpl staining were blocked with M.O.M Mouse IgG Blocking Reagent (Vector Laboratories) stained with anti Dpl GX-2D10-B1 (Dpl) or POM3 (soluble cellular PrP). Detection was achieved using both Goat anti Mouse AP and Donkey anti Goat AP (Jackson) with alkaline phosphatase fast red.

## Supporting Information

Figure S1 Characterization of transgenic mice (A) Gene copy numbers per haploid genome in transgenic lines as determined by genomic Q-PCR. (B) relative mRNA level in brain extracts of transgenes compared to PrP mRNA in C57BL/6 mice (filled black columns and left y-axis) and compared Dpl mRNA in *Prnp* ^Ngsk/Ngsk^ mice (open columns and right y-axis) using either PrP or Dpl specific primer sets for Q-PCR. Each column represents the average of 3 mice.
(0.55 MB TIF)

Figure S2 Characterization of membrane anchored and PI-PLC treated transgenic proteins. Density gradient DRM preparations of wild-type, PrP GPI anchorless (PrP_s_), PrP_ΔCD_ and PrP_ΔCDs_ transgenic brains analyzed after PI-PLC treatment and deglycosylation with PNGase F with monoclonal antibody POM1. After PI-PLC treatment PrP and PrP_ΔCD_ had similarly buoyancy like PrPs and PrP_ΔCDs_ whereas flotillin a non GPI-anchored DRM associated protein still was found in fractions with higher buoyancy indicating the intactness of the DRMs.
(0.84 MB TIF)
